# Supplementary material for: Retro-cue alerting effects on conscious perception across different levels of target visibility
Source: Psychol Res. 2026 Oct 9;90(5):184. doi: 10.1007/s00426-026-02353-y (PMC13652783; doi:10.1007/s00426-026-02353-y)
Supplement: Supplementary file 1 [file 426_2026_2353_MOESM1_ESM.pdf]

Supplementary materials

Experiment 1

Reaction times

T-tests were conducted to compare the reaction times (RTs) between Awareness conditions, showing a significant difference between Seen and Unseen trials ($t(35) = 10.715$, $p < .001$, Cohen's $d = 2.063$, $BF_{10}) = 6.153 \times 10^9$ and demonstrating that RTs were significantly faster in the Unseen condition.

The hierarchical backwards likelihood-ratio tests (LRTs) revealed a significant effect of SOA ($\chi^2(5) = 14.332$, $p < .05$). Post-hoc analyses (see Table S1) showed a trend of faster RTs when the alerting tone was presented before or simultaneous to the Gabor stimulus (see Figure S1), although posthoc comparisons did not reach significance.

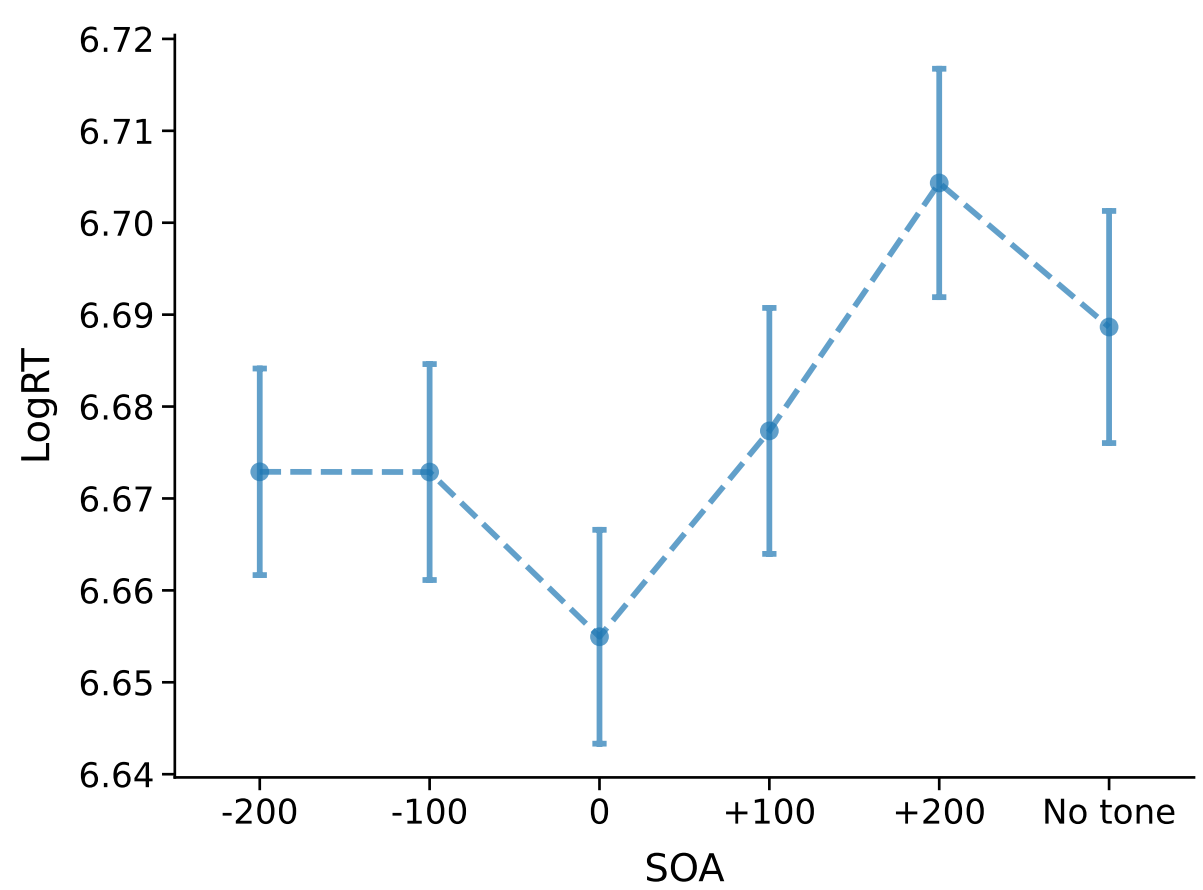

Figure S1: Reaction times for Experiment 1 as a function of SOA. Error bars represent standard errors.

Table S1: Post-hoc pairwise comparisons of RT for each level of SOA in Experiment 1.

Contrast	Estimate	CI [2.5%, 97.5%]	SE	Z-stat	P-val	Sig
(-200) - (-100)	-.005	[-.045, .035]	.014	-.359	> .05	
(-200) - (0)	.018	[-.023, .058]	.014	1.283	> .05	
(-200) - (+100)	-.009	[-.050, .033]	.014	-.600	> .05	
(-200) - (+200)	-.031	[-.073, .011]	.014	-2.146	> .05	
(-200) - (No tone)	-.025	[-.067, .017]	.014	-1.769	> .05	
(-100) - (0)	.022	[-.019, .063]	.014	1.601	> .05	
(-100) - (+100)	-.004	[-.046, .039]	.015	-.253	> .05	
(-100) - (+200)	-.026	[-.069, .017]	.015	-1.769	> .05	
(-100) - (No tone)	-.020	[-.063, .022]	.015	-1.395	> .05	
(0) - (+100)	-.026	[-.069, .017]	.015	-1.777	> .05	
(0) - (+200)	-.048	[-.092, -.005]	.015	-3.277	.016	*
(0) - (No tone)	-.043	[-.085, .000]	.015	-2.924	> .05	
(+100) - (+200)	-.022	[-.067, .023]	.015	-1.456	> .05	
(+100) - (No tone)	-.017	[-.061, .028]	.015	-1.094	> .05	
(+200) - (No tone)	.006	[-.039, .050]	.015	.375	> .05	

Note: * $p < .05$, ** $p < .01$, *** $p < .001$; CI = Confidence Interval; SE = Standard Error; Z-stat = Z statistic; P-val = P-value; Sig. = Significance level.

Accuracy

As demonstrated by the t-tests, the proportion of seen targets was higher when the response to the tilt discrimination response was correct, as compared to incorrect responses ($t(35) = 18.053$, $p < .001$, Cohen's $d = 2.894$, $BF_{10} = 1.769 \times 10^{16}$).

The linear mixed models (LMMs) revealed a significant effect of SOA on accuracy (ACC) ($\chi^2(5) = 11.604$, $p < .05$), although pairwise comparisons did not reach significance (see Table S2).

Table S2: Post-hoc pairwise comparisons of ACC for each level of SOA in Experiment 1.

Contrast	Estimate	CI [2.5%, 97.5%]	SE	Z-stat	P-val	Sig
(-200) - (-100)	.417	[-.265, 1.099]	.232	1.796	> .05	
(-200) - (0)	.041	[-.670, .752]	.242	.170	> .05	
(-200) - (+100)	-.194	[-.973, .584]	.265	-.732	> .05	
(-200) - (+200)	.263	[-.463, .990]	.247	1.065	> .05	
(-200) - (No tone)	.558	[-.150, 1.265]	.241	2.314	> .05	
(-100) - (0)	-.376	[-1.065, .313]	.235	-1.600	> .05	
(-100) - (+100)	-.611	[-1.372, .149]	.259	-2.360	> .05	
(-100) - (+200)	-.154	[-.856, .549]	.239	-.642	> .05	
(-100) - (No tone)	.140	[-.539, .820]	.232	.606	> .05	
(0) - (+100)	-.235	[-1.016, .545]	.266	-.885	> .05	
(0) - (+200)	.222	[-.508, .952]	.249	.893	> .05	
(0) - (No tone)	.516	[-.198, 1.230]	.243	2.123	> .05	
(+100) - (+200)	.458	[-.339, 1.254]	.271	1.686	> .05	
(+100) - (No tone)	.752	[-.031, 1.534]	.267	2.820	> .05	
(+200) - (No tone)	.294	[-.432, 1.020]	.247	1.189	> .05	

Note: * $p < .05$, ** $p < .01$, *** $p < .001$; CI = Confidence Interval; SE = Standard Error; Z-stat = Z statistic; P-val = P-value; Sig. = Significance level.

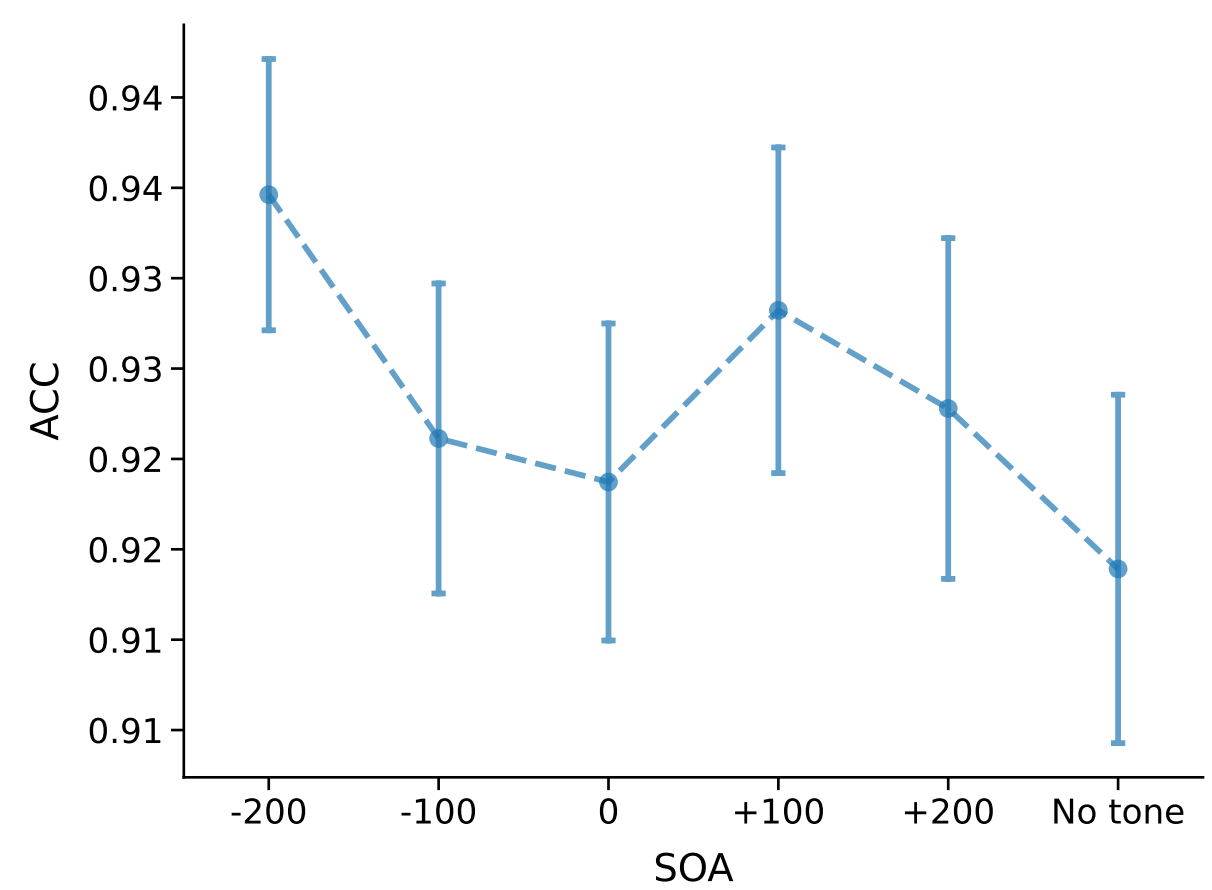

Figure S2: Accuracy for Experiment 1 as a function of SOA. Error bars represent standard errors.

Proportion seen and SDT tables

Table S3: Post-hoc pairwise comparisons of Seen for each level of SOA in Experiment 1.

Contrast	Estimate	CI [2.5%, 97.5%]	SE	Z-stat	P-val	Sig
(-200) - (-100)	.241	[-.018, .500]	.088	2.727	> .05	
(-200) - (0)	.366	[.110, .623]	.087	4.197	< .001	***
(-200) - (+100)	.611	[.352, .870]	.088	6.924	< .001	***
(-200) - (+200)	.696	[.438, .955]	.088	7.913	< .001	***
(-200) - (No tone)	.611	[.354, .868]	.088	6.983	< .001	***
(-100) - (0)	.126	[-.129, .380]	.087	1.448	> .05	
(-100) - (+100)	.370	[.113, .627]	.088	4.221	< .001	***
(-100) - (+200)	.455	[.199, .712]	.087	5.214	< .001	***
(-100) - (No tone)	.370	[.116, .625]	.087	4.267	< .001	***
(0) - (+100)	.244	[-.010, .498]	.087	2.821	> .05	
(0) - (+200)	.330	[.076, .583]	.086	3.820	.002	**
(0) - (No tone)	.245	[-.007, .496]	.086	2.851	> .05	
(+100) - (+200)	.086	[-.170, .341]	.087	.984	> .05	
(+100) - (No tone)	.000	[-.254, .254]	.087	.004	> .05	
(+200) - (No tone)	-.085	[-.338, .168]	.086	-.988	> .05	

Note: * $p < .05$, ** $p < .01$, *** $p < .001$; CI = Confidence Interval; SE = Standard Error; Z-stat = Z statistic; P-val = P-value; Sig. = Significance level.

Table S4: Post-hoc pairwise comparisons of d' for each level of SOA in Experiment 1.

Contrast	T-stat	DF	P-val	BF10	Cohen's *d*	Sig.
(-100) vs (-200)	-1.834	35	> .05	.808	-.229	
(-100) vs (0)	.631	35	> .05	.216	.081	
(-100) vs (+100)	3.892	35	.006	67.436	.486	**
(-100) vs (+200)	3.825	35	.008	56.73	.514	**
(-100) vs (No tone)	1.875	35	> .05	.863	.258	
(-200) vs (0)	2.696	35	> .05	3.991	.293	
(-200) vs (+100)	5.540	35	< .001	6009.368	.670	***
(-200) vs (+200)	5.268	35	< .001	2797.977	.709	***
(-200) vs (No tone)	3.294	35	.034	15.291	.474	*
(0) vs (+100)	3.263	35	.037	14.2	.389	*
(0) vs (+200)	3.125	35	> .05	10.293	.405	
(0) vs (No tone)	1.070	35	> .05	.304	.160	
(+100) vs (+200)	-.113	35	> .05	.18	-.015	
(+100) vs (No tone)	-2.205	35	> .05	1.52	-.262	
(+200) vs (No tone)	-2.105	35	> .05	1.269	-.270	

Note: * $p < .05$, ** $p < .01$, *** $p < .001$; CI = Confidence Interval; SE = Standard Error; Z-stat = Z statistic; P-val = P-value; Sig. = Significance level.

Experiment 2

Reaction times

First, paired-samples t-tests were conducted to compare the RTs between the seen and unseen trials. The results showed that the seen trials had significantly lower RTs ($t(34) = 7.341$, $p < .001$, Cohen's $d = 1.392$, $BF_{10} = 8.027 \times 10^5$) than the unseen trials.

The hierarchical backwards LRTs revealed a significant effect of SOA ($\chi^2(5) = 34.547$, $p < .001$).

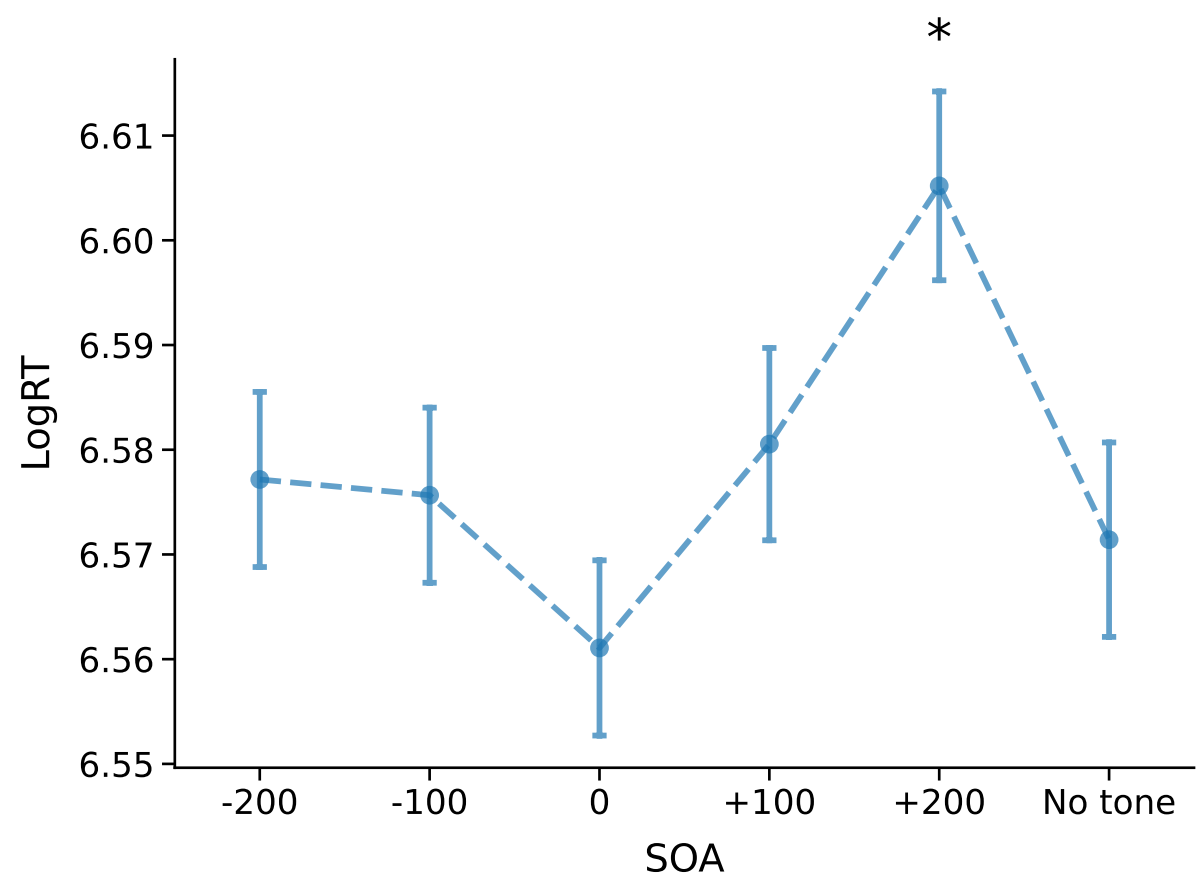

Figure S3: Reaction times (RT) across SOA conditions in Experiment 2. Error bars represent standard errors. Asterisks indicate significant post-hoc comparisons against the No tone condition

Posthoc pairwise comparisons were conducted to further explore this effect (see Table S5). This revealed that although pre-stimulus (-200 and -100 ms), simultaneous (0 ms), and post-stimulus (+100 ms) tones showed significantly faster RTs than the +200 ms condition, they were not significantly different to the no tone condition. The only significant difference was between the +200 ms and No tone conditions, demonstrating that presenting the tone 200 ms after the Gabor stimulus produced significantly slower responses.

Table S5: Post-hoc pairwise comparisons of RT for each level of SOA in Experiment 2.

Contrast	Estimate	CI [2.5%, 97.5%]	SE	Z-stat	P-val	Sig
(-200) - (-100)	.008	[-.020, .035]	.009	.821	> .05	
(-200) - (0)	.016	[-.013, .045]	.010	1.651	> .05	
(-200) - (+100)	-.008	[-.037, .022]	.010	-.756	> .05	
(-200) - (+200)	-.041	[-.071, -.011]	.010	-3.973	.001	**
(-200) - (No tone)	-.005	[-.035, .024]	.010	-.510	> .05	
(-100) - (0)	.008	[-.020, .036]	.010	.871	> .05	
(-100) - (+100)	-.015	[-.044, .013]	.010	-1.561	> .05	
(-100) - (+200)	-.049	[-.079, -.019]	.010	-4.786	< .001	***
(-100) - (No tone)	-.013	[-.042, .016]	.010	-1.299	> .05	
(0) - (+100)	-.024	[-.053, .006]	.010	-2.343	> .05	
(0) - (+200)	-.057	[-.088, -.026]	.010	-5.465	< .001	***
(0) - (No tone)	-.021	[-.051, .009]	.010	-2.079	> .05	
(+100) - (+200)	-.034	[-.065, -.002]	.011	-3.168	.023	*
(+100) - (No tone)	.002	[-.028, .033]	.010	.229	> .05	
(+200) - (No tone)	.036	[.004, .067]	.011	3.334	.013	*

Note: * $p < .05$, ** $p < .01$, *** $p < .001$; CI = Confidence Interval; SE = Standard Error; Z-stat = Z statistic; P-val = P-value; Sig. = Significance level.

Accuracy

Paired-samples t-tests revealed that the seen trials had significantly higher ACC than unseen trials ($t(34) = 16.427$, $p < .001$, Cohen's $d = 3.418$, $BF_{10} = 5.318 \times 10^{14}$).

The LMMs analysis of ACC revealed that the SOA was not a significant predictor ($\chi^2(5) = 5.728$, $p = .333$; see Figure S4).

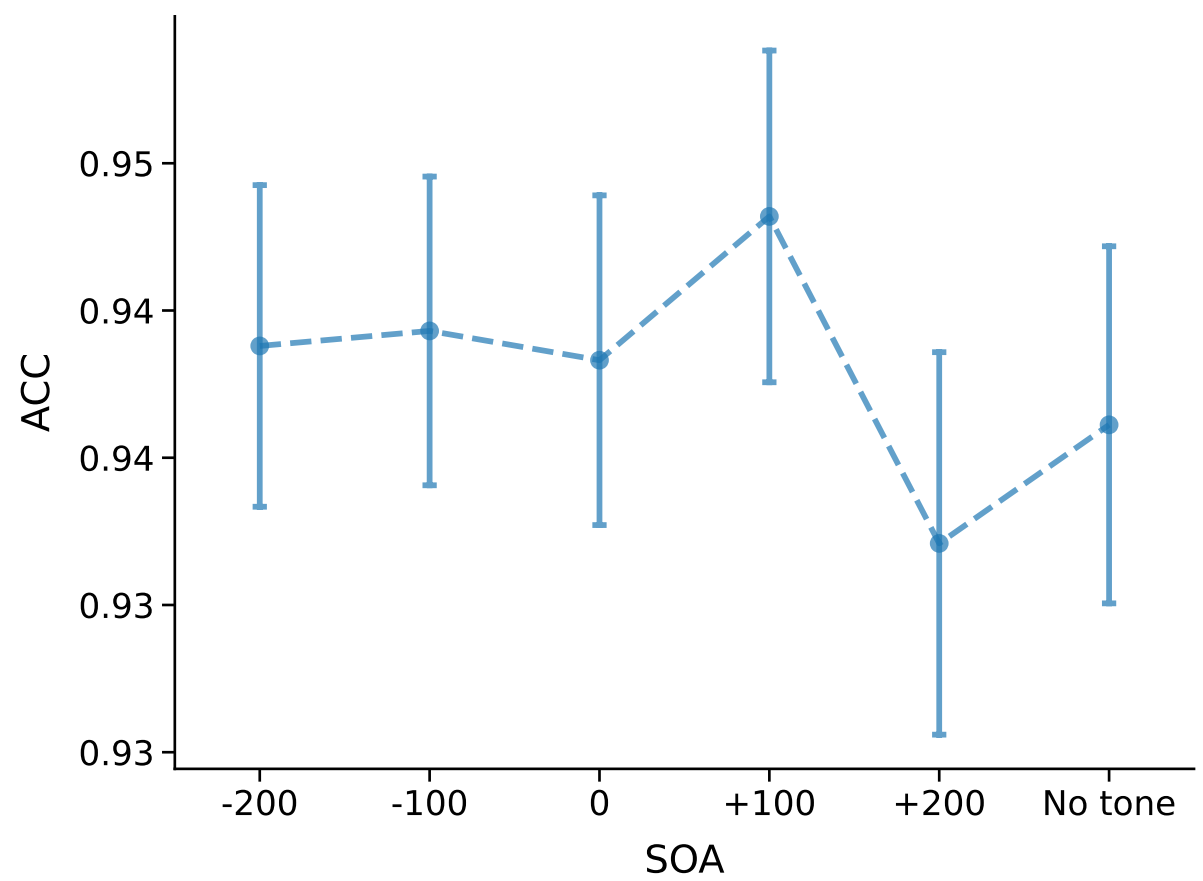

Figure S4: Accuracy across SOA and Awareness conditions in Experiment 2. Error bars represent standard errors.

Table S6: Post-hoc pairwise comparisons of ACC for each level of SOA in Experiment 2.

Contrast	Estimate	CI [2.5%, 97.5%]	SE	Z-stat	P-val	Sig
(-200) - (-100)	.173	[-.392, .738]	.238	.727	> .05	
(-200) - (0)	.049	[-.528, .625]	.242	.200	> .05	
(-200) - (+100)	-.553	[-1.157, .051]	.255	-2.167	> .05	
(-200) - (+200)	.183	[-.407, .773]	.248	.738	> .05	
(-200) - (No tone)	-.168	[-.743, .406]	.241	-.699	> .05	
(-100) - (0)	-.125	[-.682, .433]	.234	-.531	> .05	
(-100) - (+100)	-.726	[-1.314, -.138]	.247	-2.937	> .05	
(-100) - (+200)	.010	[-.559, .578]	.239	.040	> .05	
(-100) - (No tone)	-.341	[-.901, .218]	.235	-1.455	> .05	
(0) - (+100)	-.601	[-1.198, -.005]	.251	-2.396	> .05	
(0) - (+200)	.134	[-.443, .711]	.243	.552	> .05	
(0) - (No tone)	-.216	[-.783, .351]	.238	-.909	> .05	
(+100) - (+200)	.736	[.127, 1.344]	.255	2.880	> .05	
(+100) - (No tone)	.385	[-.211, .980]	.250	1.540	> .05	
(+200) - (No tone)	-.351	[-.916, .214]	.237	-1.480	> .05	

Note: * $p < .05$, ** $p < .01$, *** $p < .001$; CI = Confidence Interval; SE = Standard Error; Z-stat = Z statistic; P-val = P-value; Sig. = Significance level.

Proportion seen and SDT tables

Table S7: Post-hoc pairwise comparisons of Seen for each level of SOA in Experiment 2.

Contrast	Estimate	CI [2.5%, 97.5%]	SE	Z-stat	P-val	Sig
(-200) - (-100)	-.112	[-.297, .073]	.063	-1.776	> .05	
(-200) - (0)	.172	[-.012, .357]	.063	2.744	> .05	
(-200) - (+100)	.334	[.149, .518]	.063	5.318	< .001	***
(-200) - (+200)	.652	[.468, .835]	.063	10.412	< .001	***
(-200) - (No tone)	.424	[.239, .610]	.063	6.716	< .001	***
(-100) - (0)	.284	[.101, .467]	.062	4.557	< .001	***
(-100) - (+100)	.446	[.262, .629]	.062	7.138	< .001	***
(-100) - (+200)	.764	[.581, .947]	.062	12.243	< .001	***
(-100) - (No tone)	.536	[.352, .720]	.063	8.550	< .001	***
(0) - (+100)	.161	[-.021, .343]	.062	2.601	> .05	
(0) - (+200)	.479	[.298, .661]	.062	7.756	< .001	***
(0) - (No tone)	.252	[.069, .435]	.062	4.041	.001	***
(+100) - (+200)	.318	[.137, .499]	.062	5.151	< .001	***
(+100) - (No tone)	.091	[-.092, .273]	.062	1.458	> .05	
(+200) - (No tone)	-.227	[-.409, -.045]	.062	-3.662	.004	**

Note: * *p* < .05, ** *p* < .01, *** *p* < .001; CI = Confidence Interval; SE = Standard Error; Z-stat = Z statistic; P-val = P-value; Sig. = Significance level.

Table S8: Post-hoc pairwise comparisons of d' for each level of SOA in Experiment 2.

Contrast	T-stat	P-val	BF10	Cohen's *d*	Sig.
(-200) - (-100)	-.753	> .05	.236	-.105	
(-200) - (0)	.204	> .05	.185	.031	
(-200) - (+100)	.655	> .05	.221	.116	
(-200) - (+200)	2.616	.197	3.385	.380	
(-200) - (No tone)	2.090	.662	1.246	.357	
(-100) - (0)	.892	> .05	.262	.146	
(-100) - (+100)	1.404	> .05	.445	.236	
(-100) - (+200)	3.895	.007	66.329	.498	**
(-100) - (No tone)	3.109	> .05	9.87	.476	
(0) - (+100)	.586	> .05	.213	.095	
(0) - (+200)	2.785	> .05	4.82	.390	
(0) - (No tone)	2.066	> .05	1.194	.365	
(+100) - (+200)	2.153	> .05	1.392	.309	
(+100) - (No tone)	1.511	> .05	.51	.282	
(+200) - (No tone)	-.210	> .05	.185	-.034	

Note: * *p* < .05, ** *p* < .01, *** *p* < .001; CI = Confidence Interval; SE = Standard Error; Z-stat = Z statistic; P-val = P-value; Sig. = Significance level.

Table S9: Post-hoc pairwise comparisons of β for each level of SOA in Experiment 2.

Contrast	W-val	P-val	η^2	Sig.
(-200) - (0)	263	> .05	.002	
(-200) - (No tone)	268	> .05	.005	
(-100) - (-200)	279	> .05	.001	
(-100) - (0)	279	> .05	.005	
(-100) - (No tone)	230	> .05	.010	
(0) - (No tone)	285	> .05	.001	
(+100) - (-200)	209	> .05	.020	
(+100) - (-100)	190	> .05	.031	
(+100) - (0)	240	> .05	.010	
(+100) - (+200)	289	> .05	.000	
(+100) - (No tone)	239	> .05	.005	
(+200) - (-200)	228	> .05	.016	
(+200) - (-100)	167	> .05	.026	
(+200) - (0)	238	> .05	.007	
(+200) - (No tone)	274	> .05	.003	

Note: * $p < .05$, ** $p < .01$, *** $p < .001$; CI = Confidence Interval; SE = Standard Error; Z-stat = Z statistic; P-val = P-value; Sig. = Significance level.

Experiment 3

Reaction times

T-tests showed significant differences in RTs across Awareness levels, with responses in the seen trials being significantly slower than in the unseen trials ($t(34) = 14.425$, $p < .001$, Cohen's $d = 2.831$, $BF_{10} = 1.272 \times 10^{13}$).

For the LMMs, backwards LRTs revealed a significant effect of SOA (χ^2 (5) = 721.790, $p < .001$), with responses being significantly faster when the tone was presented before or simultaneous to the target onset as shown in Figure S5, although no pairwise comparisons agains the No tone condition were significant (see table S10).

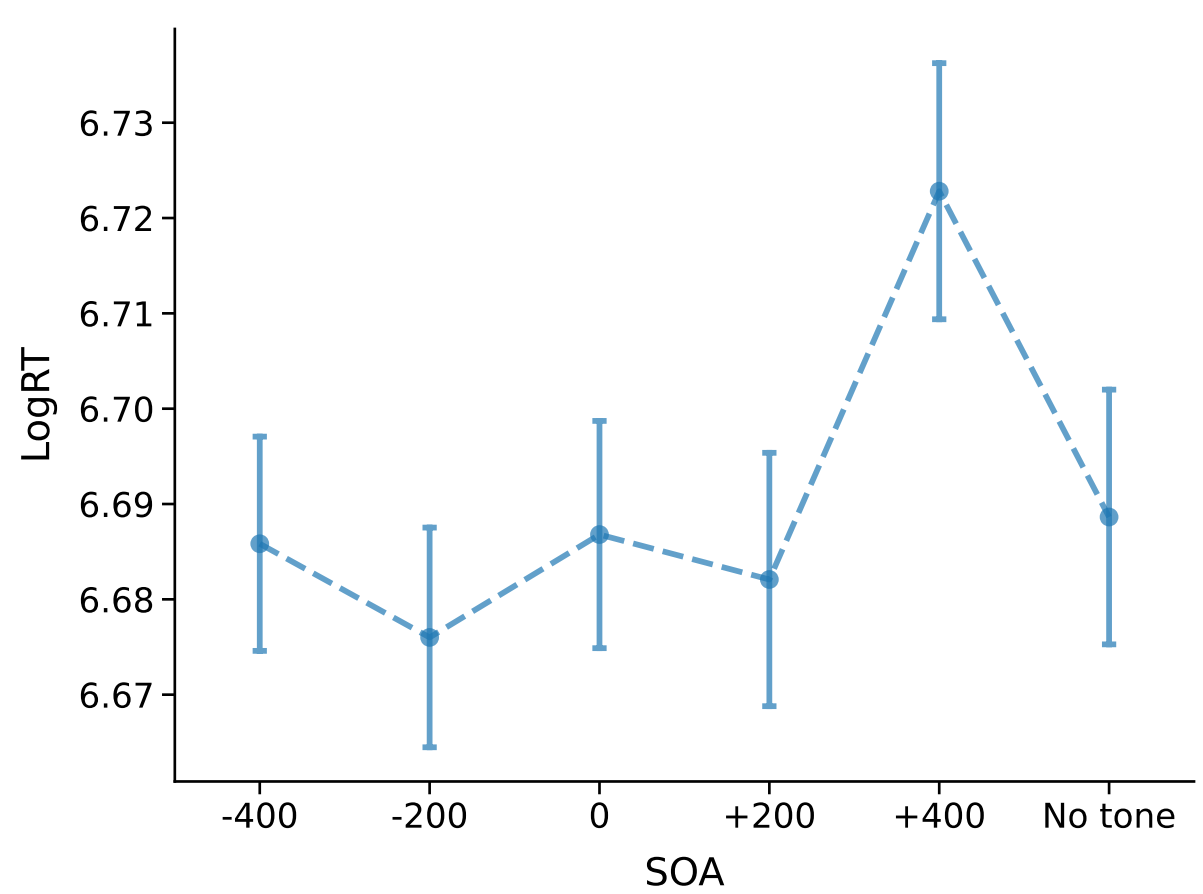

Figure S5: Reaction times (RT) across SOA conditions in Experiment 3. Error bars represent standard errors.

Accuracy

The paired-samples t-test showed significant differences in ACC across Awareness levels, with higher accuracy in the seen trials compared to unseen trials ($t(34) = 30.678$, $p < .001$, Cohen's $d = 6.089$, $BF_{10} = 1.16 \times 10^{23}$).

In the LMM analysis, the backwards LRTs found no significant effect of SOA (χ^2 (5) = 8.191, $p > .05$).

Table S10: Post-hoc pairwise comparisons of RT for each level of SOA in Experiment 3.

Contrast	Estimate	CI [2.5%, 97.5%]	SE	T-stat	P-val	Sig
(-400) - (-200)	.010	[-.028, .047]	.013	.749	> .05	
(-400) - (0)	-.003	[-.043, .036]	.013	-.260	> .05	
(-400) - (+200)	.011	[-.031, .052]	.014	.757	> .05	
(-400) - (+400)	-.047	[-.090, -.005]	.014	-3.305	.014	*
(-400) - (No tone)	-.023	[-.064, .019]	.014	-1.623	> .05	
(-200) - (0)	-.013	[-.052, .026]	.013	-.976	> .05	
(-200) - (+200)	.001	[-.040, .043]	.014	.078	> .05	
(-200) - (+400)	-.057	[-.099, -.015]	.014	-3.952	.001	**
(-200) - (No tone)	-.032	[-.074, .009]	.014	-2.292	> .05	
(0) - (+200)	.014	[-.029, .057]	.015	.964	> .05	
(0) - (+400)	-.044	[-.088, -.000]	.015	-2.951	.048	*
(0) - (No tone)	-.019	[-.062, .024]	.015	-1.323	> .05	
(+200) - (+400)	-.058	[-.104, -.012]	.016	-3.730	.003	**
(+200) - (No tone)	-.034	[-.079, .011]	.015	-2.190	> .05	
(+400) - (No tone)	.025	[-.021, .070]	.016	1.581	> .05	

Note: * $p < .05$, ** $p < .01$, *** $p < .001$; CI = Confidence Interval; SE = Standard Error; Z-stat = Z statistic; P-val = P-value; Sig. = Significance level.

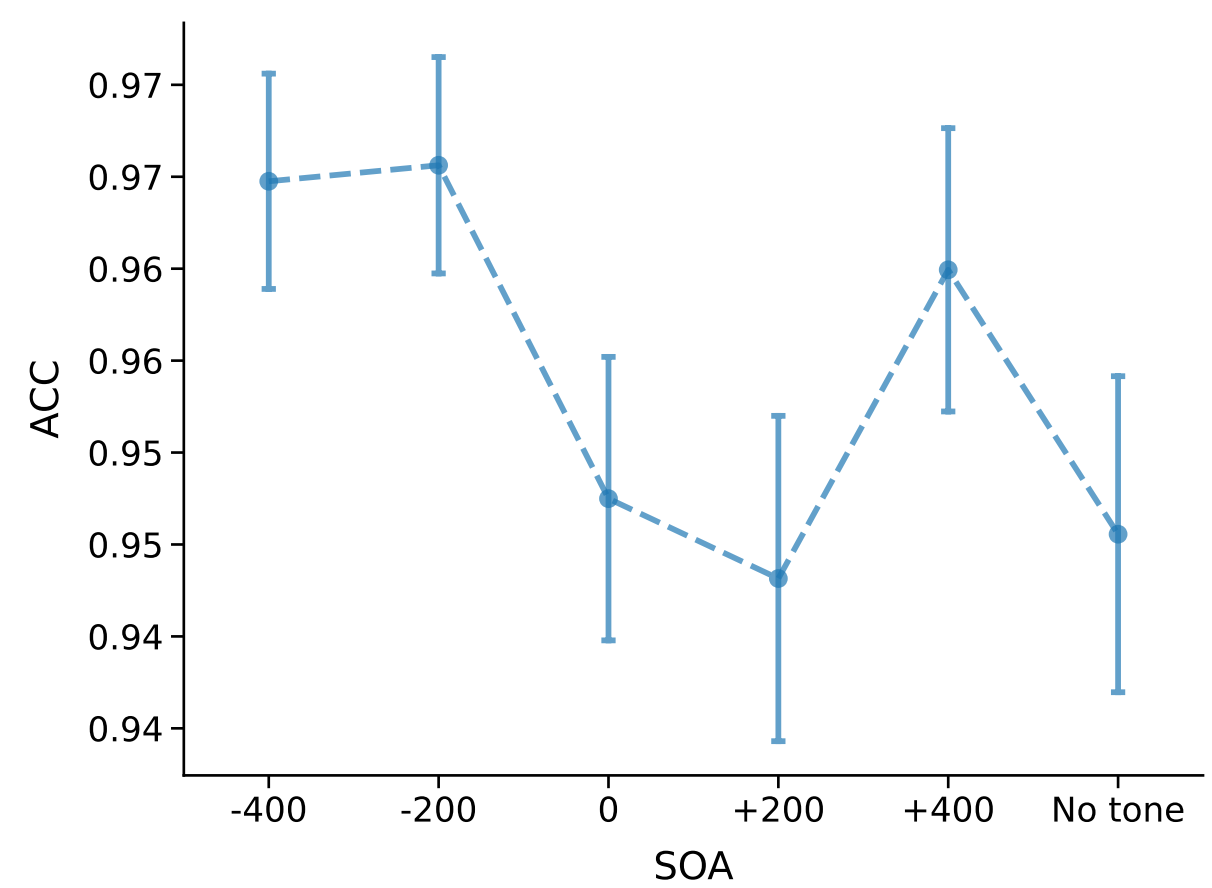

Figure S6: ACC across SOA conditions in Experiment 3. Error bars represent standard errors.

Table S11: Post-hoc pairwise comparisons of ACC for each level of SOA in Experiment 3.

Contrast	Estimate	CI [2.5%, 97.5%]	SE	Z-stat	P-val	Sig
(-400) - (-200)	-.034	[-.768, .700]	.250	-.137	> .05	
(-400) - (0)	.319	[-.376, 1.014]	.237	1.346	> .05	
(-400) - (+200)	.452	[-.266, 1.170]	.245	1.847	> .05	
(-400) - (+400)	-.052	[-.842, .739]	.269	-.192	> .05	
(-400) - (No tone)	.396	[-.326, 1.118]	.246	1.611	> .05	
(-200) - (0)	.353	[-.351, 1.056]	.240	1.472	> .05	
(-200) - (+200)	.486	[-.242, 1.214]	.248	1.961	> .05	
(-200) - (+400)	-.018	[-.817, .782]	.272	-.065	> .05	
(-200) - (No tone)	.430	[-.300, 1.161]	.249	1.729	> .05	
(0) - (+200)	.133	[-.554, .821]	.234	.570	> .05	
(0) - (+400)	-.370	[-1.132, .391]	.260	-1.427	> .05	
(0) - (No tone)	.078	[-.613, .768]	.235	.330	> .05	
(+200) - (+400)	-.504	[-1.287, .279]	.267	-1.889	> .05	
(+200) - (No tone)	-.056	[-.768, .657]	.243	-.230	> .05	
(+400) - (No tone)	.448	[-.338, 1.234]	.268	1.674	> .05	

Note: * $p < .05$, ** $p < .01$, *** $p < .001$; CI = Confidence Interval; SE = Standard Error; Z-stat = Z statistic; P-val = P-value; Sig. = Significance level.

Proportion seen and SDT tables

Table S12: Post-hoc pairwise comparisons of Seen for each level of SOA in Experiment 3.

Contrast	Estimate	CI [2.5%, 97.5%]	SE	Z-stat	P-val	Sig
(-400) - (-200)	.067	[-.206, .340]	.093	.717	> .05	
(-400) - (0)	.426	[.157, .694]	.091	4.655	< .001	***
(-400) - (+200)	.816	[.545, 1.087]	.092	8.833	< .001	***
(-400) - (+400)	.846	[.573, 1.118]	.093	9.106	< .001	***
(-400) - (No tone)	.827	[.558, 1.096]	.092	9.018	< .001	***
(-200) - (0)	.359	[.092, .626]	.091	3.942	.001	**
(-200) - (+200)	.749	[.479, 1.019]	.092	8.145	< .001	***
(-200) - (+400)	.779	[.508, 1.051]	.092	8.424	< .001	***
(-200) - (No tone)	.760	[.492, 1.028]	.091	8.330	< .001	***
(0) - (+200)	.390	[.125, .655]	.090	4.324	< .001	***
(0) - (+400)	.420	[.154, .687]	.091	4.629	< .001	***
(0) - (No tone)	.401	[.138, .664]	.090	4.478	< .001	***
(+200) - (+400)	.030	[-.238, .298]	.091	.332	> .05	
(+200) - (No tone)	.011	[-.253, .276]	.090	.125	> .05	
(+400) - (No tone)	-.019	[-.285, .247]	.091	-.210	> .05	

Note: * $p < .05$, ** $p < .01$, *** $p < .001$; CI = Confidence Interval; SE = Standard Error; Z-stat = Z statistic; P-val = P-value; Sig. = Significance level.

Table S13: Post-hoc pairwise comparisons of d' for each level of SOA in Experiment 3.

Contrast	W-val	P-val	η^2	Sig.
(-400) - (0)	152.000	.100	.040	
(-400) - (No tone)	32.000	.000	.160	***
(-200) - (-400)	287.000	1.000	.001	
(-200) - (0)	132.000	.209	.028	
(-200) - (No tone)	32.000	.000	.142	***
(0) - (No tone)	88.000	.001	.078	**
(+200) - (-400)	64.000	.000	.091	***
(+200) - (-200)	70.000	.000	.075	***
(+200) - (0)	209.000	1.000	.022	
(+200) - (+400)	275.000	1.000	.000	
(+200) - (No tone)	190.000	.606	.018	
(+400) - (-400)	70.000	.000	.107	***
(+400) - (-200)	99.000	.003	.089	**
(+400) - (0)	180.000	.395	.028	
(+400) - (No tone)	219.000	1.000	.021	

Note: * $p < .05$, ** $p < .01$, *** $p < .001$; CI = Confidence Interval; SE = Standard Error; Z-stat = Z statistic; P-val = P-value; Sig. = Significance level.

Table S14: Post-hoc pairwise comparisons of β for each level of SOA in Experiment 3.

Contrast	W-val	P-val	η^2	Sig.
(-400) - (0)	253.000	1.000	.001	
(-400) - (No tone)	204.000	1.000	.014	
(-200) - (-400)	249.000	1.000	.007	
(-200) - (0)	247.000	1.000	.002	
(-200) - (No tone)	172.000	.274	.038	
(0) - (No tone)	193.000	.685	.024	
(+200) - (-400)	258.000	1.000	.007	
(+200) - (-200)	289.000	1.000	.000	
(+200) - (0)	304.000	1.000	.002	
(+200) - (+400)	290.000	1.000	.000	
(+200) - (No tone)	140.000	.051	.042	
(+400) - (-400)	260.000	1.000	.011	
(+400) - (-200)	309.000	1.000	.000	
(+400) - (0)	309.000	1.000	.004	
(+400) - (No tone)	157.000	.130	.054	

Note: * $p < .05$, ** $p < .01$, *** $p < .001$; CI = Confidence Interval; SE = Standard Error; Z-stat = Z statistic; P-val = P-value; Sig. = Significance level.

Experiment 4

Reaction times

First, we compared RTs for seen versus unseen trials by means of a t-test, which revealed significantly faster RTs for seen trials compared to unseen trials ($t(34) = 9.772$, $p < .001$, Cohen's $d = 1.526$, $BF_{10} = 4.476 \times 10^8$).

For the LMMs, backwards LRTs revealed a significant main effect of SOA (χ^2 (5) = 39.615, $p < .001$), with faster responses when the tone was presented before or simultaneously with the target, compared to when the tone was presented after the target (see Figure S7).

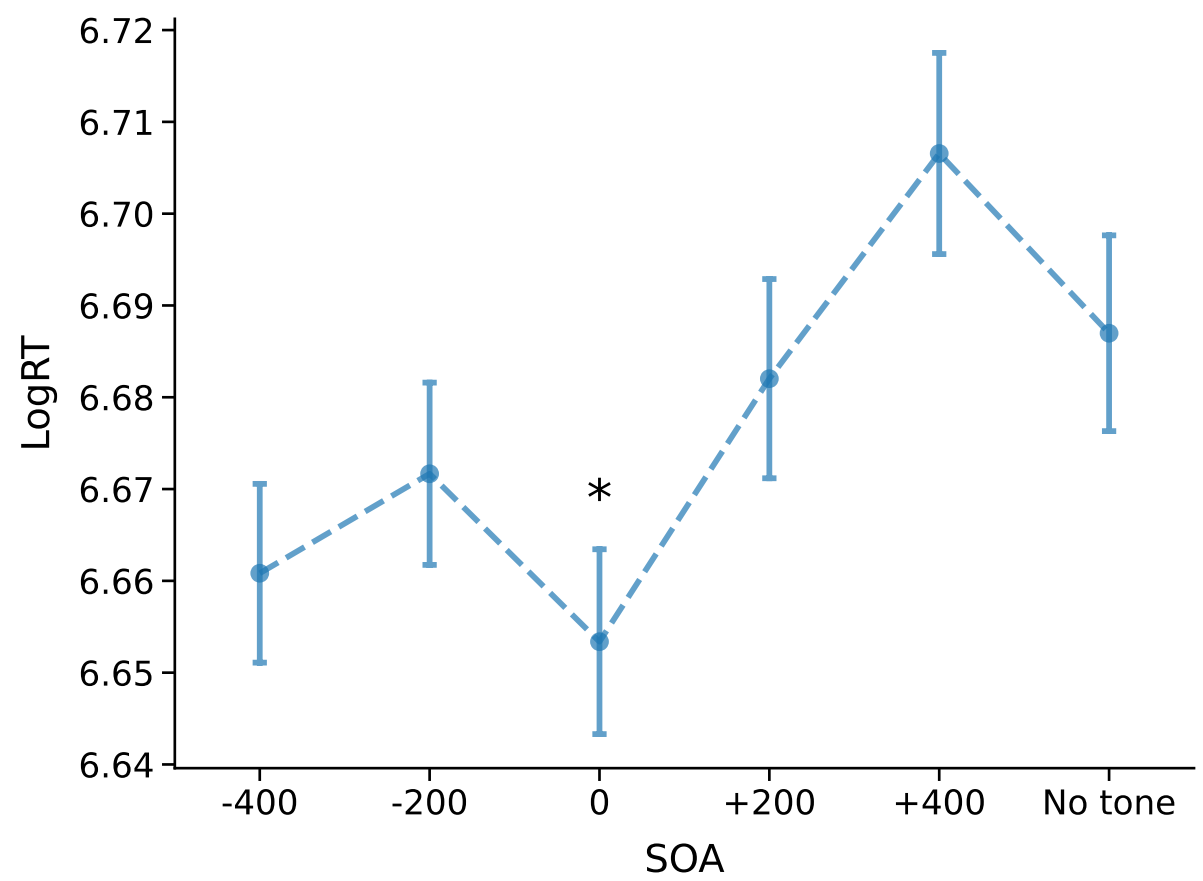

Figure S7: RTs across SOA conditions in Experiment 4. Error bars represent standard errors. Asterisks indicate significant post-hoc comparisons against the No tone condition

Table S15: Post-hoc pairwise comparisons of RT for each level of SOA in Experiment 4.

Contrast	Estimate	CI [2.5%, 97.5%]	SE	T-stat	P-val	Sig
(-400) - (-200)	-.007	[-.037, .023]	.010	-.723	> .05	
(-400) - (0)	.010	[-.021, .041]	.011	.950	> .05	
(-400) - (+200)	-.026	[-.058, .006]	.011	-2.403	> .05	
(-400) - (+400)	-.052	[-.084, -.020]	.011	-4.716	< .001	***
(-400) - (No tone)	-.024	[-.057, .008]	.011	-2.220	> .05	
(-200) - (0)	.017	[-.013, .048]	.010	1.676	> .05	
(-200) - (+200)	-.019	[-.050, .013]	.011	-1.749	> .05	
(-200) - (+400)	-.045	[-.077, -.013]	.011	-4.091	.001	***
(-200) - (No tone)	-.017	[-.049, .015]	.011	-1.565	> .05	
(0) - (+200)	-.036	[-.069, -.004]	.011	-3.281	.016	*
(0) - (+400)	-.062	[-.095, -.029]	.011	-5.554	< .001	***
(0) - (No tone)	-.034	[-.067, -.002]	.011	-3.092	.030	*
(+200) - (+400)	-.026	[-.060, .008]	.012	-2.239	> .05	
(+200) - (No tone)	.002	[-.032, .036]	.011	.161	> .05	
(+400) - (No tone)	.028	[-.006, .062]	.012	2.385	> .05	

Note: * $p < .05$, ** $p < .01$, *** $p < .001$; CI = Confidence Interval; SE = Standard Error; Z-stat = Z statistic; P-val = P-value; Sig. = Significance level.

Accuracy

Paired-samples t-tests comparing accuracy between seen and unseen trials revealed significantly higher accuracy for seen trials compared to unseen trials ($t(34) = 23.786$, $p < .001$, Cohen's $d = 5.130$, $BF_{10} = 3.852 \times 10^{19}$).

For the LMM analysis, backwards LRTs revealed a significant main effect of SOA (χ^2 (5) = 12.220, $p = .031$). Post-hoc pairwise comparisons indicated a general effect of the tone increasing the ACC when compared to no presenting the tone, regardless of the SOA (see Figure S8 and Table S16).

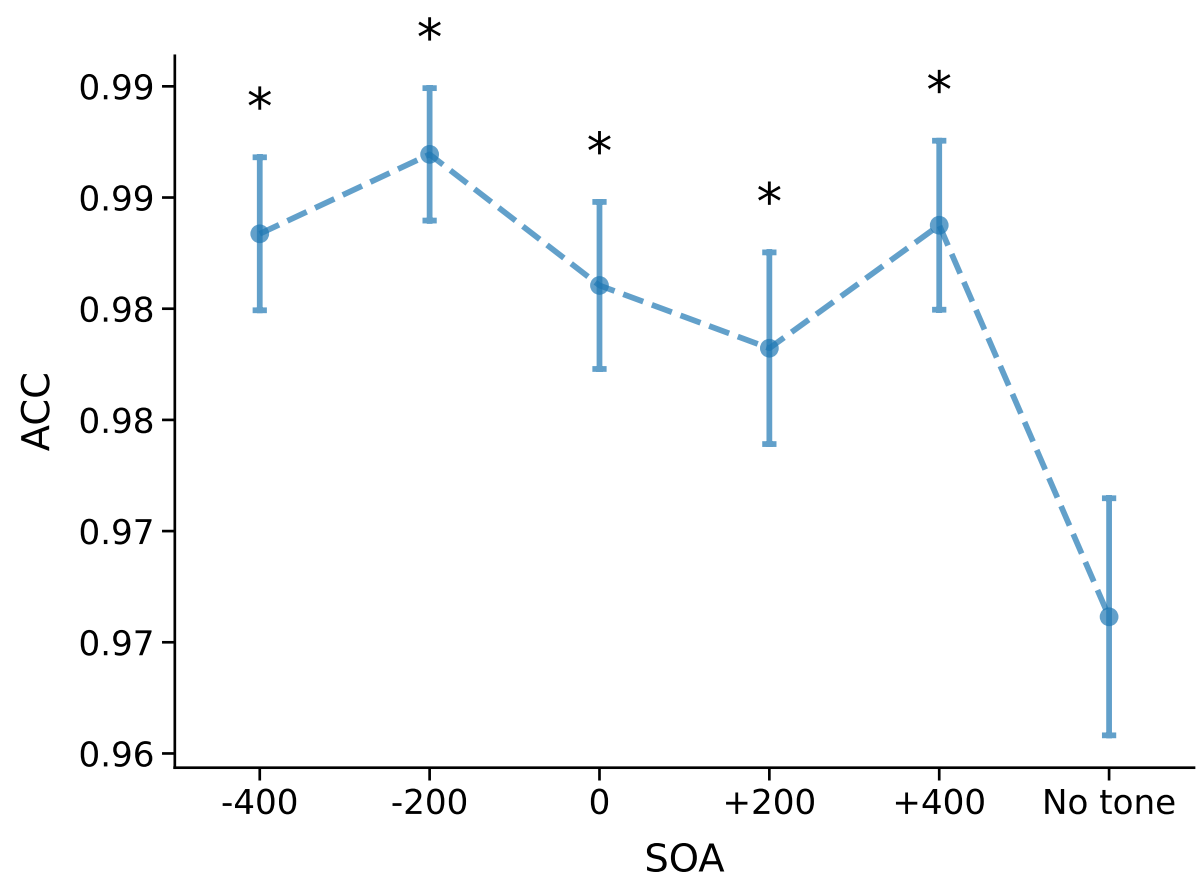

Figure S8: ACC across SOA conditions in Experiment 4. Error bars represent standard errors. Asterisks indicate significant post-hoc comparisons against the No tone condition.

Table S16: Post-hoc pairwise comparisons of ACC for each level of SOA in Experiment 4.

Contrast	Estimate	CI [2.5%, 97.5%]	SE	Z-stat	P-val	Sig
(-400) - (-200)	-.276	[-.279, -.274]	.001	-324.098	< .001	***
(-400) - (0)	.221	[.219, .224]	.001	259.226	< .001	***
(-400) - (+200)	.209	[.207, .212]	.001	245.547	< .001	***
(-400) - (+400)	-.105	[-.108, -.103]	.001	-123.446	< .001	***
(-400) - (No tone)	.706	[.703, .708]	.001	826.984	< .001	***
(-200) - (0)	.498	[.494, .501]	.001	412.480	< .001	***
(-200) - (+200)	.486	[.482, .490]	.001	402.808	< .001	***
(-200) - (+400)	.171	[.168, .175]	.001	141.885	< .001	***
(-200) - (No tone)	.982	[.978, .986]	.001	813.954	< .001	***
(0) - (+200)	-.012	[-.015, -.008]	.001	-9.681	< .001	***
(0) - (+400)	-.326	[-.330, -.323]	.001	-270.597	< .001	***
(0) - (No tone)	.484	[.481, .488]	.001	401.456	< .001	***
(+200) - (+400)	-.315	[-.318, -.311]	.001	-260.922	< .001	***
(+200) - (No tone)	.496	[.492, .500]	.001	411.146	< .001	***
(+400) - (No tone)	.811	[.807, .814]	.001	672.068	< .001	***

Note: * $p < .05$, ** $p < .01$, *** $p < .001$; CI = Confidence Interval; SE = Standard Error; Z-stat = Z statistic; P-val = P-value; Sig. = Significance level.

Proportion seen and SDT tables

Table S17: Post-hoc pairwise comparisons of Seen for each level of SOA in Experiment 4.

Contrast	Estimate	CI [2.5%, 97.5%]	SE	Z-stat	P-val	Sig
(-400) - (-200)	-.138	[-.522, .246]	.131	-1.054	> .05	
(-400) - (0)	.378	[.022, .734]	.121	3.120	.027	*
(-400) - (+200)	.721	[.375, 1.068]	.118	6.112	< .001	***
(-400) - (+400)	.981	[.646, 1.317]	.114	8.588	< .001	***
(-400) - (No tone)	1.027	[.694, 1.359]	.113	9.066	< .001	***
(-200) - (0)	.516	[.154, .877]	.123	4.190	< .001	***
(-200) - (+200)	.859	[.507, 1.212]	.120	7.159	< .001	***
(-200) - (+400)	1.119	[.778, 1.461]	.116	9.614	< .001	***
(-200) - (No tone)	1.165	[.826, 1.503]	.115	10.094	< .001	***
(0) - (+200)	.343	[.023, .664]	.109	3.142	.025	*
(0) - (+400)	.604	[.295, .913]	.105	5.733	< .001	***
(0) - (No tone)	.649	[.343, .954]	.104	6.231	< .001	***
(+200) - (+400)	.260	[-.038, .558]	.102	2.563	> .05	
(+200) - (No tone)	.305	[.011, .600]	.100	3.043	.035	*
(+400) - (No tone)	.045	[-.236, .327]	.096	.471	> .05	

Note: * $p < .05$, ** $p < .01$, *** $p < .001$; CI = Confidence Interval; SE = Standard Error; Z-stat = Z statistic; P-val = P-value; Sig. = Significance level.

Table S18: Post-hoc pairwise comparisons of d' for each level of SOA in Experiment 4.

Contrast	T-stat	P-val	BF10	Cohen's *d*	Sig.
(-400) - (-200)	-.491	>.05	.203	-.090	
(-400) - (0)	1.120	>.05	.323	.249	
(-400) - (+200)	2.663	>.05	3.729	.463	
(-400) - (+400)	3.229	.041	13.008	.640	*
(-400) - (No tone)	4.730	.001	601.236	.900	***
(-200) - (0)	2.258	>.05	1.684	.348	
(-200) - (+200)	3.299	.034	15.334	.588	*
(-200) - (+400)	3.532	.018	26.834	.763	*
(-200) - (No tone)	5.699	.000	8628.595	1.042	***
(0) - (+200)	.842	>.05	.252	.179	
(0) - (+400)	2.021	>.05	1.106	.375	
(0) - (No tone)	3.170	.048	11.339	.626	*
(+200) - (+400)	1.121	>.05	.323	.244	
(+200) - (No tone)	3.127	>.05	10.27	.541	
(+400) - (No tone)	1.420	>.05	.454	.258	

Note: * $p < .05$, ** $p < .01$, *** $p < .001$; CI = Confidence Interval; SE = Standard Error; Z-stat = Z statistic; P-val = P-value; Sig. = Significance level.

Table S19: Post-hoc pairwise comparisons of β for each level of SOA in Experiment 4.

Contrast	W-val	P-val	η^2	Sig.
(-400) - (0)	225	>.05	.024	
(-400) - (No tone)	71	.000	.184	***
(-200) - (-400)	280	>.05	.002	
(-200) - (0)	169	>.05	.040	
(-200) - (No tone)	29	.000	.210	***
(0) - (No tone)	129	>.05	.095	
(+200) - (-400)	80	.001	.181	***
(+200) - (-200)	40	.000	.210	***
(+200) - (0)	124	.019	.083	*
(+200) - (+400)	254	>.05	.009	
(+200) - (No tone)	268	>.05	.002	
(+400) - (-400)	46	.000	.224	***
(+400) - (-200)	57	.000	.250	***
(+400) - (0)	83	.004	.126	**
(+400) - (No tone)	281	>.05	.002	

Note: * $p < .05$, ** $p < .01$, *** $p < .001$; CI = Confidence Interval; SE = Standard Error; Z-stat = Z statistic; P-val = P-value; Sig. = Significance level.

Comparison between Experiment 3 and Experiment 4

To more directly assess whether the retro-cue effect depended on target visibility, an additional between-experiments comparison was conducted including Experiments 3 and 4, which used the same SOAs but differed in target visibility (50% vs. 75%). Specifically, a combined mixed-effects model was fitted with Experiment and SOA as fixed factors:

$$Seen \sim SOA \times Experiment + (1|Participant)$$

This analysis revealed a strong main effect of Experiment, with higher overall seen rates in Experiment 4 (.857) than in Experiment 3 (.644), consistent with the higher visibility range introduced by the titration procedure. Importantly, there was also a small but significant SOA $\times$ Experiment interaction ($\chi^2(5) = 11.36$, $p = .045$), indicating that the modulation of seen responses across SOAs differed between the two experiments.

To further examine whether the retro-cue benefit itself differed across experiments, an index of retro-cue benefit was computed for each SOA by subtracting the proportion of seen responses in the No tone condition from the proportion of seen responses in each SOA condition. This index was then directly compared between Experiments 3 and 4. In this analysis, the SOA $\times$ Experiment interaction did not reach significance ($p = .060$). A planned comparison at the +200 ms SOA showed a numerically larger retro-cue benefit in Experiment 4 than in Experiment 3, but this difference did not reach significance ($p = .235$). Table S20 reports the pairwise contrasts (Experiment 3 vs Experiment 4) for each SOA, including confidence intervals and test statistics.

Taken together, these supplementary analyses provide converging but not definitive support for the visibility-dependent interpretation of the retro-cue effect. Because this between-experiments comparison was not planned *a priori* and the study was not powered specifically for this contrast, these results should be interpreted cautiously as trend-level support rather than as conclusive evidence.

Table S20: Pairwise contrasts for the comparison between Experiment 3 and Experiment 4 across SOAs, in the proportion of seen index.

SOA	Estimate	CI [2.5%, 97.5%]	SE	DF	T-stat	P-val
-400	.0660	[.0180, .1140]	.0240	306.58	2.69	.008
-200	.0460	[-.0030, .0940]	.0240	306.58	1.86	.064
0	.0320	[-.0160, .0800]	.0240	306.58	1.31	.193
$+200$	-.0290	[-.0770, .0190]	.0240	306.58	-1.19	.235
$+400$	.0190	[-.0290, .0670]	.0240	306.58	0.77	.440

Note: CI = Confidence Interval; SE = Standard Error; DF = Degrees of Freedom; T-stat = T statistic; P-val = P-value.
